# Supplementary material for: Longitudinal clinical proteomics reveals pneumonia type-specific protein biomarkers and autoantibodies
Source: medRxiv. 2026 Jan 13:2026.01.12.26343938. Preprint. [Version 1] doi: 10.64898/2026.01.12.26343938 (PMC12870487; doi:10.64898/2026.01.12.26343938)
Supplement: Supplement 1 [file NIHPP2026.01.12.26343938v1-supplement-1.pdf]

medRxiv preprint doi: https://doi.org/10.64898/2026.01.12.26343938; this version posted January 13, 2026. The copyright holder for this preprint (which was not certified by peer review) is the author/funder, who has granted medRxiv a license to display the preprint in perpetuity.
It is made available under a CC-BY 4.0 International license .

Supplementary Figures

Figure S1

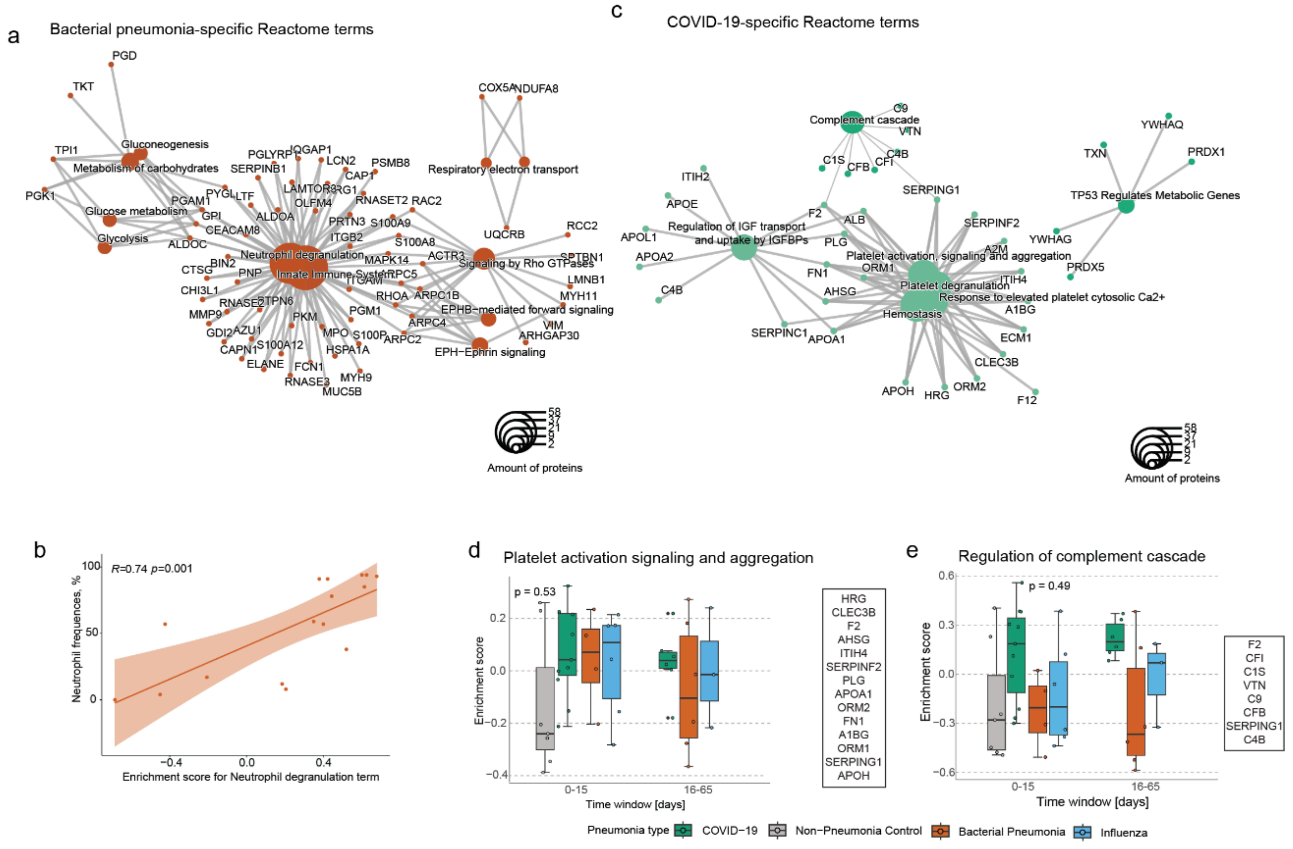

Figure S1 - related to Figure 1. (a-b) Network plot of representative (a) bacterial-specific and (b) COVID-19-specific BALF protein modules. The circle size of the terms corresponds to the number of included proteins. (c) Pearson correlation of neutrophil degranulation proteins and normalized neutrophil frequency in the BALF of bacterial pneumonia patients. (d-e) Box plots displaying the longitudinal enrichment of (d) platelet activation signaling and aggregation and (e) regulation of complement cascade Reactome term proteins in BALF specimens across the four pneumonia types. Each dot represents the enrichment score for an individual patient. Data are represented as mean ± SD and were statistically assessed with the non-parametric Kruskal-Wallis test. Proteins involved in the term are displayed on the right side of the plot.

medRxiv preprint doi: https://doi.org/10.64898/2026.01.12.26343938; this version posted January 13, 2026. The copyright holder for this preprint (which was not certified by peer review) is the author/funder, who has granted medRxiv a license to display the preprint in perpetuity.
It is made available under a CC-BY 4.0 International license .

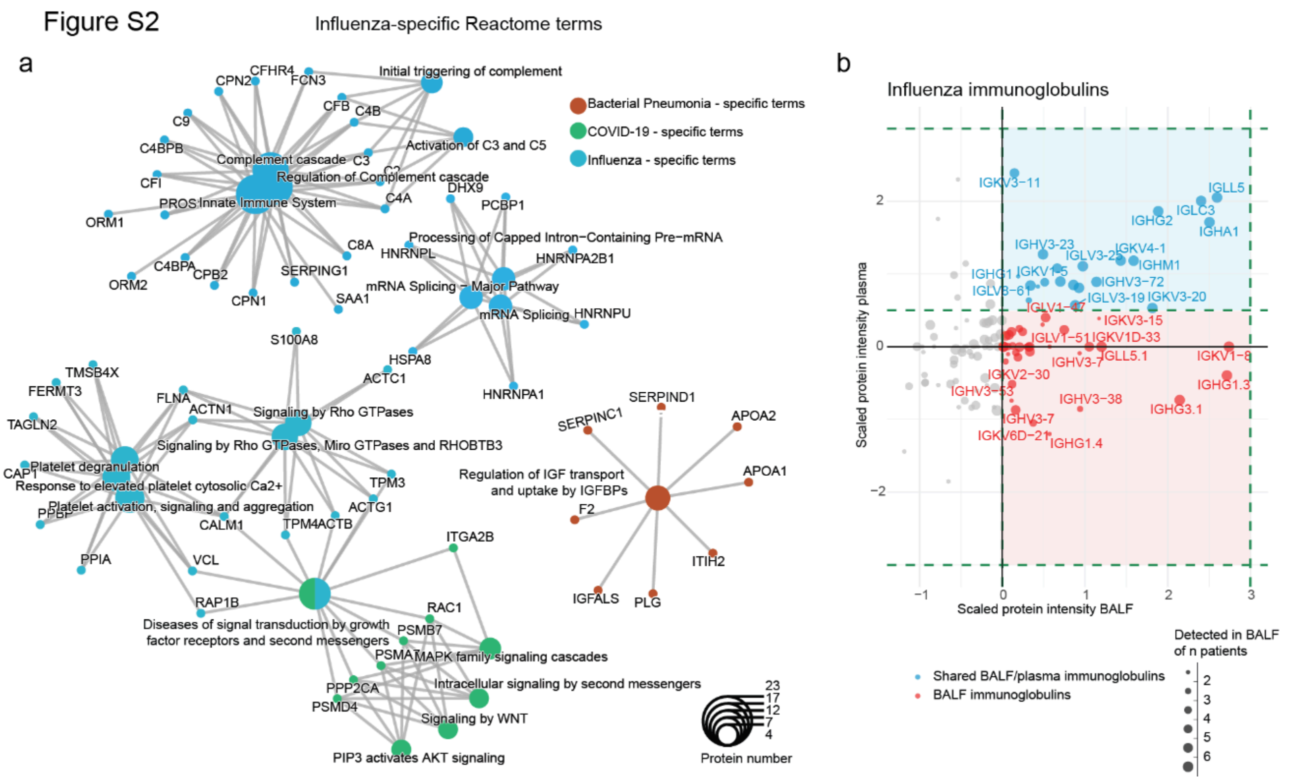

Figure S2 - related to Figure 2. (a) Network plots of representative bacterial-specific, COVID-19-specific and influenza-specific plasma protein modules. The circle size of the terms corresponds to the number of included proteins. (b) Scatter plot displaying immunoglobulin segment abundance in the BALF (x-axis) and plasma (y-axis) of Influenza patients at the time point upon intubation. Dot size corresponds to the number of patients that express a detected protein.

medRxiv preprint doi: https://doi.org/10.64898/2026.01.12.26343938; this version posted January 13, 2026. The copyright holder for this preprint (which was not certified by peer review) is the author/funder, who has granted medRxiv a license to display the preprint in perpetuity.
It is made available under a CC-BY 4.0 International license .

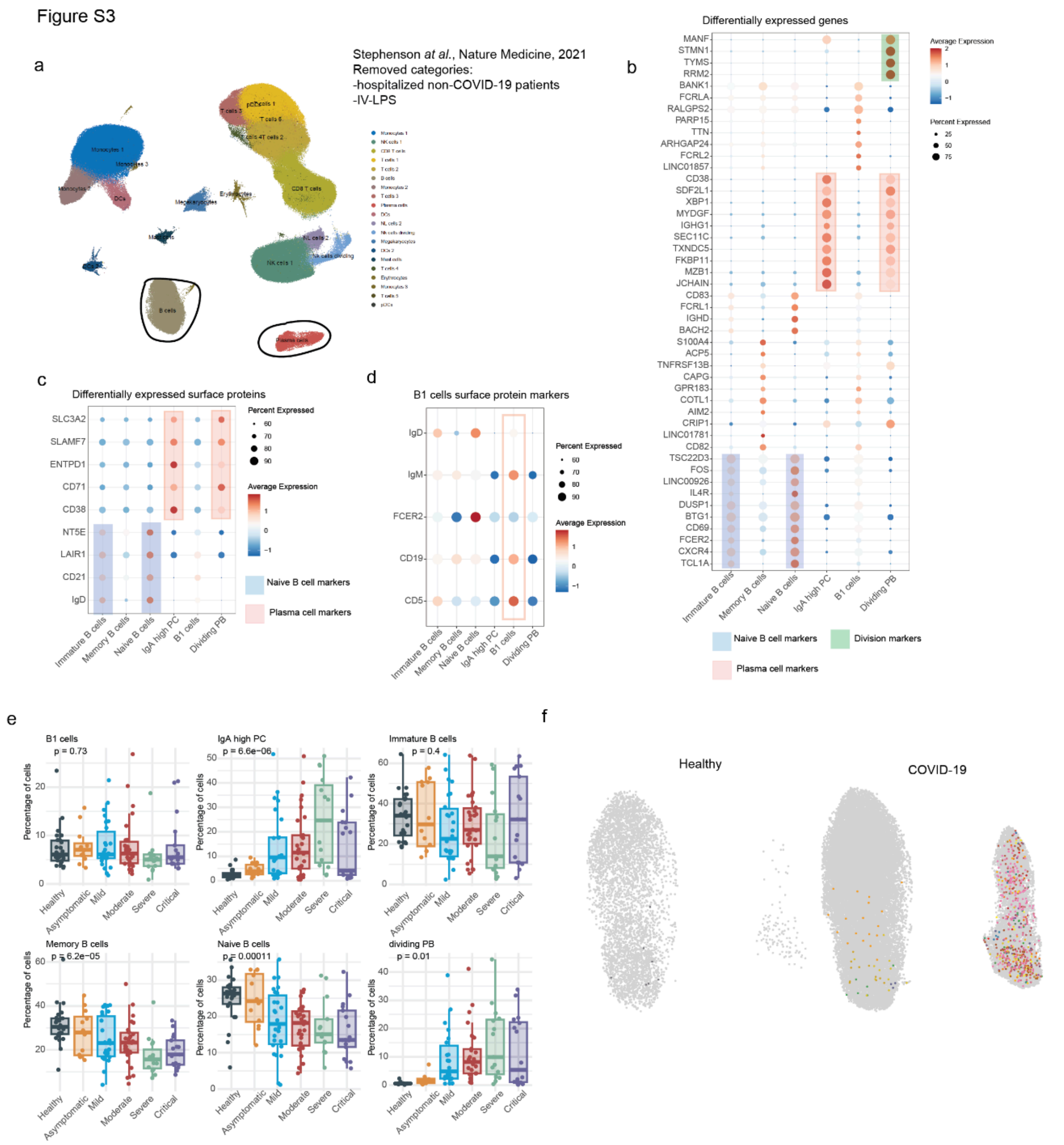

Figure S3 - related to Figure 3. (a) UMAP plot of 632.209 PBMCs from 90 COVID-19 patients and 23 healthy controls, colored by annotation [24]. B and plasma cell clusters are circled. (b) Dot plot of the top 10 unique gene markers for each identified B cell cluster. (c) Dot plot of differentially expressed surface protein markers for each identified B cell cluster. (d) Dot plot of B1 B cell-specific surface protein markers for each identified B cell cluster. (e) Bar plots showing the isotype distribution of B and plasma cell populations across COVID-19 disease severity groups. (f) Box plots showing the proportion of B and plasma cell populations across COVID-19 disease severity groups. Each dot represents an individual patient sample. Data are represented as mean ± SD and were statistically assessed with the non-parametric Kruskal-Wallis test. (g) UMAP visualization of 38,063 peripheral blood B/plasma cells from 90 COVID-19 patients and 23 healthy controls, colored by the expression of proteomics-identified V-segment clonotypes.

medRxiv preprint doi: https://doi.org/10.64898/2026.01.12.26343938; this version posted January 13, 2026. The copyright holder for this preprint (which was not certified by peer review) is the author/funder, who has granted medRxiv a license to display the preprint in perpetuity.
It is made available under a CC-BY 4.0 International license .

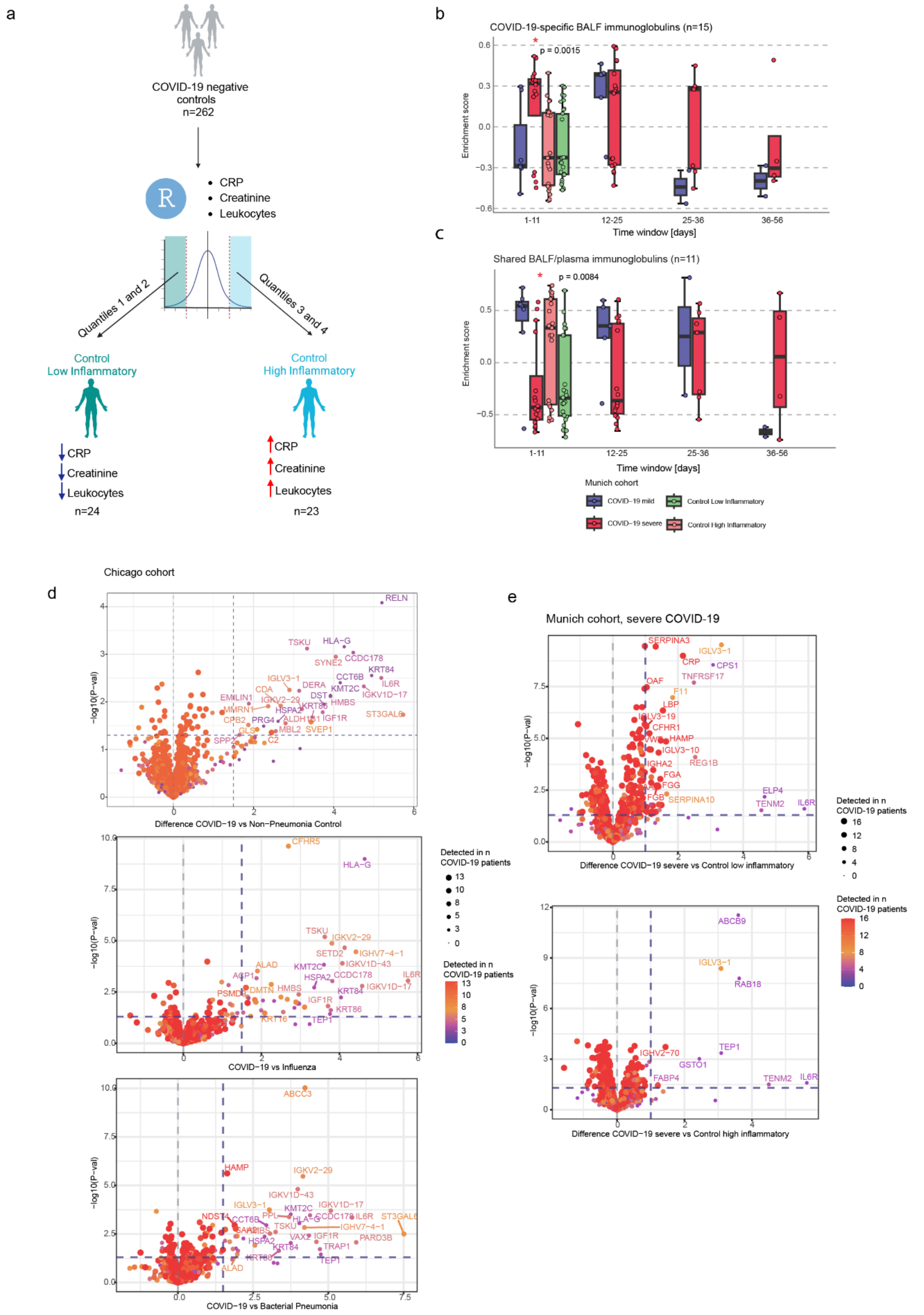

Figure S4 - related to Figure 4. (a) Schema exhibiting the criteria of selection of control low inflammatory (n=24) and control high inflammatory (n=23) groups from SARS-CoV-2 negative patients (n=264) enrolled in the University Hospital of LMU. (b-c) Box plots displaying the longitudinal enrichment of (b) BALF and (e) shared BALF/plasma segments in

medRxiv preprint doi: https://doi.org/10.64898/2026.01.12.26343938; this version posted January 13, 2026. The copyright holder for this preprint (which was not certified by peer review) is the author/funder, who has granted medRxiv a license to display the preprint in perpetuity.
It is made available under a CC-BY 4.0 International license .

BALF specimens across the four pneumonia types. Each dot represents the enrichment score for an individual patient. Data are represented as mean ± SD and were statistically assessed with the non-parametric Kruskal-Wallis test. (d-e) Volcano plots displaying immunoglobulin segment abundance between pneumonia types and controls in (d) the Chicago and (e) the Munich cohort at the time point upon intubation. Dot size corresponds to the number of patients that express a detected protein.

medRxiv preprint doi: https://doi.org/10.64898/2026.01.12.26343938; this version posted January 13, 2026. The copyright holder for this preprint (which was not certified by peer review) is the author/funder, who has granted medRxiv a license to display the preprint in perpetuity.
It is made available under a CC-BY 4.0 International license .

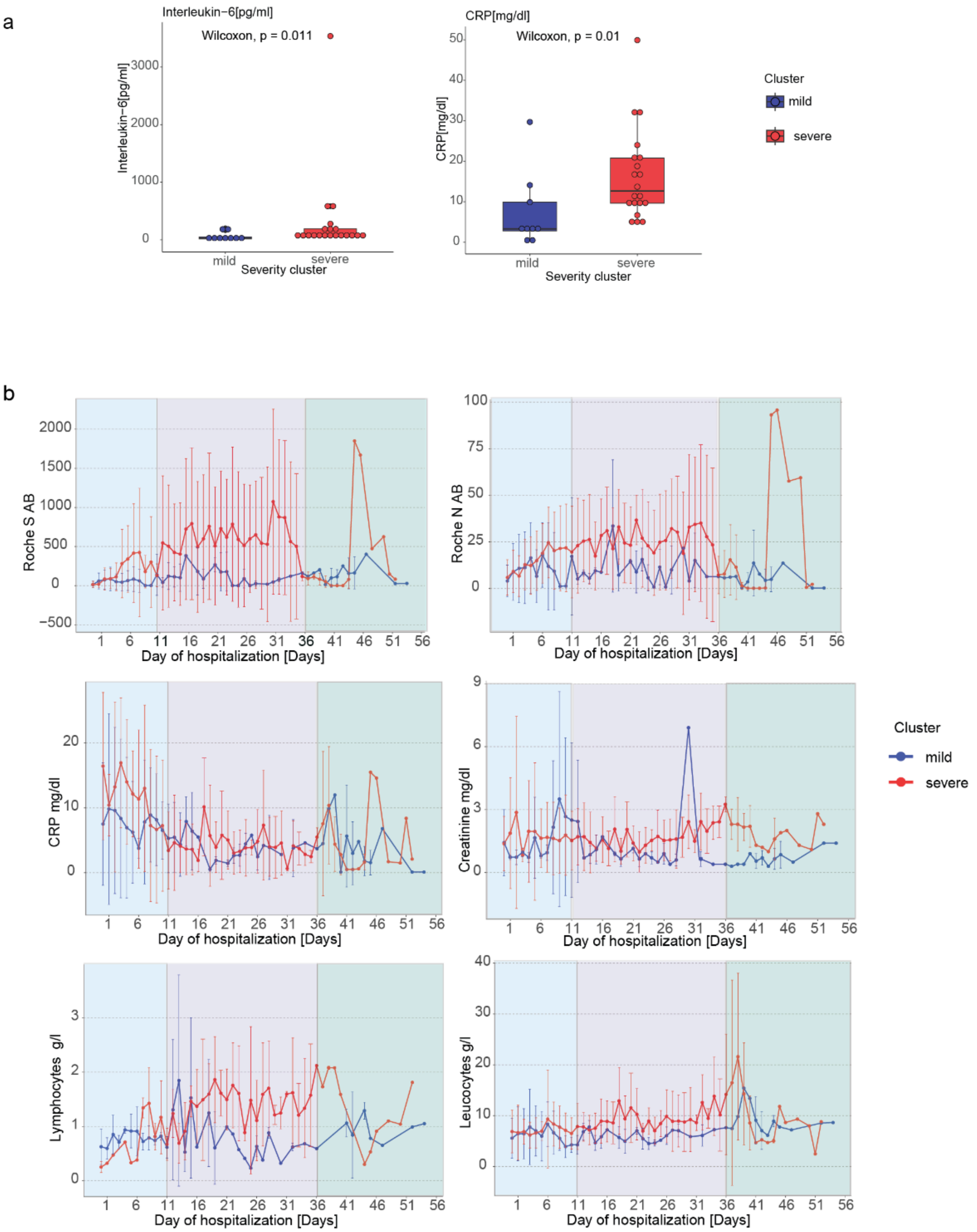

Figure S5 - related to Figure 5. (a) Secretion levels of circulating IL-6 and C-reactive protein in the two COVID-19 patient clusters of the Munich cohort. (b) Longitudinal assessment of clinical parameters for the two COVID-19 patient clusters of the Munich cohort. Three time windows were subsequently defined: 0-11 days, 12-36 days, 37-56 days.

medRxiv preprint doi: https://doi.org/10.64898/2026.01.12.26343938; this version posted January 13, 2026. The copyright holder for this preprint (which was not certified by peer review) is the author/funder, who has granted medRxiv a license to display the preprint in perpetuity.
It is made available under a CC-BY 4.0 International license .

Figure S6

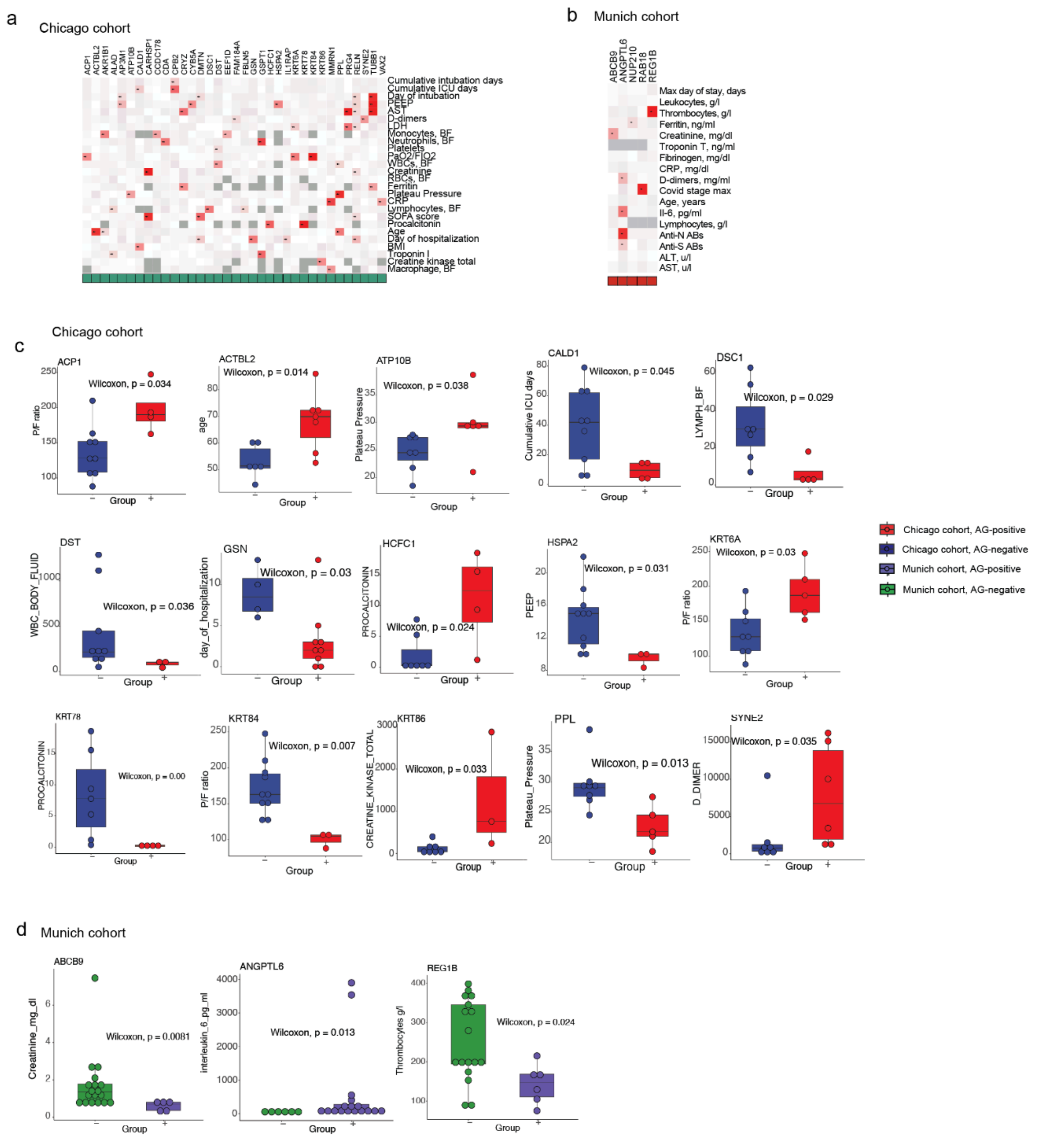

Figure S6 - related to Figure 6. (a-b) Heatmaps presenting associations between the detection of cohort-specific putative autoantibodies in the peripheral blood of (a) COVID-19 patients (n=13) of the Chicago and (b) severe COVID-19 patients (n=16) of the Munich cohort upon intubation on the x-axis and selected clinical parameters on the y-axis. The color denotes the output of the Wilcoxon test (p-value). The dark grey color corresponds to the absence of the test results due to missing values. (c-d) Box plots showing the significant associations between putative shared autoantigens and clinical parameters in two cohorts. The x-axis represents COVID-19 patients categorized based on the presence (+) or absence (-) of detected autoantigens at the time of intubation. Statistical significance was assessed using the Wilcoxon test.
